# Implementation of a Novel Epidemiological Surveillance System for Children’s Mental Health and Well-Being in France: Protocol for the National “Enabee” Cross-Sectional Study

**DOI:** 10.2196/57584

**Published:** 2024-08-13

**Authors:** Yvon Motreff, Maude Marillier, Abdessattar Saoudi, Charlotte Verdot, Louise Seconda, Damien Pognon, Imane Khireddine-Medouni, Jean-Baptiste Richard, Viviane Kovess-Masfety, Richard Delorme, Valentina Decio, Anne-Laure Perrine, Maria El Haddad, Anne Gallay, Stéphanie Monnier-Besnard, Nolwenn Regnault, Enabee Study Group

**Affiliations:** 1 Direction des maladies non transmissibles et traumatismes Santé publique France Saint-Maurice France; 2 Direction appui, traitements et analyses des données Santé publique France Saint-Maurice France; 3 Direction des régions Santé publique France Saint-Maurice France; 4 Department of Child and Adolescent Psychiatry Assistance publique - Hôpitaux de Paris (APHP) Paris Cité University Paris France; 5 See Acknowledgments Paris France

**Keywords:** child, mental health, epidemiological surveillance, well-being

## Abstract

**Background:**

Children’s mental health, including their well-being, is a major public health concern, as the burden of related disorders may last throughout one’s life. Although epidemiological mental health surveillance systems for children and adolescents have been implemented in several countries, they are sorely lacking in France.

**Objective:**

This study aims to describe the first step of the implementation of a novel surveillance system in France called Enabee (*Etude nationale sur le bien-être des enfants*), which focuses on the issue of mental health in children. The system aims to (1) describe the temporal trends in the population-based prevalence of the main mental health disorders and well-being in children aged 3 to 11 years, (2) explore their major determinants, and (3) assess mental health care use by this population. To do this, Enabee will rely on results from a recurrent national cross-sectional homonymous study. This paper presents the protocol for the first edition of this study (called Enabee 2022), as well as initial results regarding participation.

**Methods:**

Enabee 2022 is a national cross-sectional study that was implemented in French schools in 2022. It used a probabilistic, multistage, stratified, and balanced sampling plan as follows: first, schools were randomly drawn and stratified according to the type of school. Up to 4 classes per school were then randomly drawn, and finally, all the pupils within each class were selected. The study covered children from preschool and kindergarten (aged 3 to 6 years, US grading system) to fifth grade (aged 6 to 11 years). Children from first to fifth grades provided a self-assessment of their mental health using 2 validated self-administered questionnaires: the Dominic Interactive (DI) and the KINDL. Parents and teachers completed a web-based questionnaire, including the Strengths and Difficulties Questionnaire. Parents also answered additional questions about their parenting attitudes; their own mental health; known social, economic, and environmental determinants of mental health in children; and their child’s life habits. Health, education, and family stakeholders were involved in designing and implementing the study as part of a large consultation group.

**Results:**

Data were collected from May 2, 2022, to July 31, 2022, in 399 schools across metropolitan France. Teachers completed questionnaires for 5721 pupils in preschool and kindergarten and for 15,263 pupils from first to fifth grades. Parents completed questionnaires for 3785 children in preschool and kindergarten and for 9227 children from first to fifth grades. Finally, 15,206 children from first to fifth grades completed the self-administered questionnaire.

**Conclusions:**

Enabee 2022 constitutes the first milestone in the development of a novel national epidemiological surveillance system, paving the way for improved children’s mental health policies in France.

## Introduction

### Background

Children’s mental health, which in this work is considered to include well-being, is a major public health concern. Mental health disorders in children are frequent yet often left untreated: a meta-analysis of 41 studies conducted between 1985 and 2012 in 27 countries worldwide suggested that the burden of mental health disorders among children and adolescents was significant. The same meta-analysis found that worldwide, the pooled prevalence of mental disorders among children aged up to 18 years was 13.4% (95% CI 11.3%-15.9%), with the following prevalence rates for specific disorders: 6.5% (95% CI 4.7%-9.1%) for any anxiety disorder, 5.7% (95% CI 4.0%-8.1%) for any disruptive disorder, 3.4% (95% CI 2.6%-4.5%) for attention-deficit/hyperactivity disorder (ADHD), and 2.6% (95% CI 1.7%-3.9%) for any depressive disorder [1]. The School Children Mental Health in Europe project, which was implemented in 2010 in 7 countries, showed that 9.9% (ranging from 5.8% in Italy to 14.4% in Lithuania) of children required some sort of mental health care and that 76% of this proportion did not visit any mental health professional [2].

Moreover, the literature highlights that between 5% and 15% of children experience learning disorders [3,4]. These can occur in parallel with other neurodevelopmental disorders, such as ADHD [3,4]. Mental health in childhood is associated with physical [5] and mental health [6] outcomes in adulthood. In the United States, the National Comorbidity Survey Replication (NCS-R) found that half of all lifetime mental health disorders had already started by the age of 14 years [7].

Children’s mental health is closely associated with their social, economic, relational, and local environments [8,9]. Access to basic social needs for the family, parental health, and parenting attitudes, as well as life experiences both in the home and the community, are driven by social determinants of health [10]. This means that environmental conditions in the places where children live, learn, and play have a crucial impact on mental health. Children’s mental health has also been associated with other determinants, such as prenatal or perinatal factors [11,12], lifestyle habits [13], the presence of somatic chronic disease [14], parenting attitudes [15], parent’s mental health [16], and childhood adversity [17].

Even before the COVID-19 pandemic, some countries had already implemented the epidemiological surveillance of children’s and adolescents’ mental health, including the United States and New Zealand [18,19]. In England, repeated related cross-sectional studies on children’s mental health together with follow-up studies were conducted between 1999 and 2023 [20-24]. In Germany, between 2003 and 2017, the surveillance of mental health among children and adolescents was based on the cohort study Bella. Bella was based on a representative subsample of families with children from the National Health Interview and Examination Survey Among Children and Adolescents [25]. Thanks to these different preexisting monitoring systems, researchers worldwide were able to document the impact of the COVID-19 pandemic on children’s mental health during and shortly after the associated lockdowns [26-30]. Results showed that this impact was significant. For example, in England, an increase in the prevalence of any probable mental health disorder was observed between 2017 and 2020. In Germany, a comparison between data from the Bella study and data collected during the COVID-19 pandemic showed that children and adolescents experienced significantly lower health-related quality of life and more mental health problems during the pandemic [30]. These findings highlighted the necessity for each country to continuously monitor child mental health to enhance public health policies.

Data on the mental health of children in France are scarce and rely mainly on cohort studies [16,31-35] and studies of specific populations [36-38]. To date, only 2 local (ie, nonnational level) studies on mental health have been conducted in France in school-aged children, one in 1987 [39] and the other in 2004 [40]. Until now, the surveillance of child and adolescent mental health in France has relied on syndromic surveillance from emergency department data [41] and on surveys partially exploring mental health in children aged >11 years [42-45]. Given this context, it was not possible in France to monitor the impact of the COVID-19 pandemic or related mitigation strategies on the prevalence of the main mental health disorders or well-being in primary school–aged children (ie, aged 3-11 years). This limitation accelerated the development of a novel national surveillance system for the mental health of children.

In this context, a multidisciplinary group of experts was mobilized to design and implement an epidemiological surveillance system called the “National Study on Children’s Well-Being” or Enabee (*Etude nationale sur le bien-être des enfants*), which consists of repeated national cross-sectional homonymous studies.

The Enabee surveillance system aims to (1) describe the temporal trends in the population-based prevalence of the main mental health disorders and well-being in children aged 3 to 11 years, (2) explore their major determinants, and (3) assess mental health care use by this population.

### Objectives

Enabee 2022 is the first edition of the Enabee surveillance system’s recurrent national cross-sectional study. The objective of this paper is to describe the study protocol as well as provide initial results regarding participation.

## Methods

### Study Design

Enabee 2022 is a school-based, cross-sectional study focusing on children aged 3 to 11 years from metropolitan France. Conducted in 2022, it mainly used self-administered web-based questionnaires completed by parents or caregivers, teachers, and children from first to fifth grades themselves during a dedicated survey session at school.

The study population comprised children enrolled in public schools or private schools in France under contract with the French Ministry of Education. Education is mandatory from 3 to 16 years of age in the country. In 2022, a total of 99% of children (ie, 6,664,300 pupils) aged from 3 to 11 years attended these types of schools. Preschool in France for children aged 3 to 6 years has 3 tiers called *petite*, *moyenne*, and *grande*, which correspond to preschool, preschool or prekindergarten, and kindergarten, respectively, in the US system for the same age group. Elementary school for pupils aged 6 to 11 years comprises 6 levels called *CP*, *CE 1*, *CE2*, *CM1*, and *CM2*, which correspond to first to fifth grades in the US system. Children in preschool and elementary school are educated by teachers in groups (ie, classes). Specialized schools that work exclusively with children with disability were not included in Enabee 2022, nor were children schooled at home or those attending private schools not under contract with the French Ministry of Education.

### Selection of Schools and Children

We used the databases of the Ministry of Education as the survey frame. The sampling plan was designed to ensure national-level data. The plan had to take into account a number of constraints. Specifically, it had to (1) limit the number of participating schools to alleviate study costs and logistics, (2) limit the number of classes surveyed and the number of teachers solicited for the same reasons, (3) limit the cluster effect by including enough classes and schools, and (4) ensure sufficient power to specifically explore the mental health of pupils in highly socially deprived areas. To best satisfy these constraints, a probabilistic multistage stratified sampling plan was used. This first involved randomly selected schools. Up to 4 classes per school were then randomly selected. Finally, pupils within each class were selected. In schools with ≤4 classes, all the classes were selected.

We also chose to stratify the schools randomly selected according to the type of school (ie, public schools not located in highly socially deprived areas, public schools located in highly socially deprived areas, and private schools under contract with the French Ministry of Education). This stratification was used to deliberately overrepresent pupils in highly socially deprived areas. Balanced sampling was performed in the first sampling level (ie, school) on the following variables related to school specificities: age-related subtypes of schools (preschool and kindergarten: aged 3 to 6 years, elementary: aged 6 to 11 years, and primary: aged 3 to 11 years), the size of the school by quartile of the number of pupils, the median income of the commune where the school was located by quartile, the typology of the town or city where the school was located (suburb, town or city center, rural town, or isolated town), the French Deprivation Index (Fdep) [46] in quintiles, and the number of pupils by grade.

### Participation Solicitation

After the random selection of the schools, we contacted each school principal to obtain the school’s agreement to participate in the study and to schedule appointments for the dedicated survey sessions with children in the school. Posters and flyers were sent to participating schools, and school principals and the teachers of the selected classes were invited to participate in web-based meetings for further information. In parallel, parents of the children in the selected classes were informed by postal mail about the study. Parents could refuse to participate via mail, email, or phone. A child was considered as a participant if there was no active opposition from his or her parents. However, participating children could themselves refuse to participate on the day of the session. Children whose parents did not receive information about the study at home (eg, undelivered envelopes) were excluded from the study.

Data collection was conducted at the end of the school year, between May and July 2022, for two following primary reasons: (1) to ensure that the youngest children expected to self-administer the study’s child questionnaire (see the section *Child Questionnaire*), that is, those in the first grade, were capable of reading the questionnaire and (2) to ensure that teachers knew their pupils well enough to properly answer the questions related to the latter’s mental health.

Parents and teachers were asked to preferentially answer a self-administered questionnaire (see the sections *Caregiver Questionnaire* and *Teacher Questionnaire*) using a secured link on the study website. Both parents could separately fill out the questionnaire if they wished. Several reminders were sent via SMS text message, email, phone call, and postal mail from May to July 2022 to maximize teacher and parent participation. During the last phone-based reminder, parents were systematically invited to answer the questionnaire via phone. This choice was made to ensure that nonparticipation up to that call was not due to low-level digital literacy or limited computer access.

### Multiple Informants to Improve Study Validity and Robustness

The study’s protocol was designed to have several informants for the same pupil. Previous work shows that having multiple informants provides greater robustness and validity of mental health assessment than having a single informant, both in clinical practice and in epidemiological studies [47,48]. For children from first to fifth grades (aged 6 to 11 years), assessment was based on the self-administered questionnaire and the caregiver and teacher questionnaires. For children in preschool or kindergarten, assessment was based on the caregiver and teacher questionnaires.

### Child Questionnaire

#### Overview

All first- to fifth-grade children enrolled in Enabee 2022 self-administered a questionnaire that took between 20 and 30 minutes to complete. This questionnaire was downloaded on tablets with headphones and completed during school time in dedicated sessions with trained facilitators. This questionnaire comprised 2 validated instruments: the Dominic Interactive (DI) and the KINDL, which are described in the following subsections.

#### The DI Instrument

The DI is a computerized, Diagnostic and Statistical Manual of Mental Disorders, Fifth Edition–based pictorial self-administered questionnaire for children aged 6 to 11 years [49]. Children must first select an avatar according to their gender representation and appearance to facilitate identification with the character and the situations displayed in the DI. Pictures illustrate situations featuring the emotional and behavioral symptoms related to DSM-5 disorders, while a voice-over describes them. Children have to respond “yes” or “no” as to whether they share the same feeling or have the same behaviors as those of the avatar [50] (Multimedia Appendix 1). The DI comprises 81 questions screening symptoms related to 7 main mental health disorders in children (ADHD, oppositional defiant disorder, conduct disorder, major depressive disorder, separation anxiety disorder, generalized anxiety disorder, and specific phobias) and 10 questions related to strengths and competencies. The French European version has been validated [51]. Each disorder is categorized as “likely absent,” “possible,” or “probable,” according to the answers given. In our study, 13 items exploring conduct disorder in children were removed, following feedback from the study’s stakeholders, with the agreement of the DI creator. Moreover, 3 questions about exposure to peer violence, racketeering, and fear of being assaulted by peers, which were developed for a previous study conducted in 2004 in France, were added [52].

#### The KINDL Instrument

The KINDL scale assesses health-related quality of life. It comprises 24 questions, with each using a 5-point Likert answer scale (never, seldom, sometimes, often, and all the time). It explores the following 6 dimensions of well-being: physical well-being, emotional well-being, self-esteem, family, friends, and everyday functioning (school). For this study, the 6 items of the optional module on the health-related quality of life for children with chronic illness were included. The KINDL was first developed and validated in German [53,54] and was subsequently translated into several languages, including French. For the purpose of Enabee 2022, the KINDL was modified for tablet use, whereby a voice-over was added for each of the questionnaire items and the 5 possible answers (Multimedia Appendix 2).

### Teacher Questionnaire

For each pupil, teachers had to fill in a web-based questionnaire, which included the French version of the Strengths and Difficulties Questionnaire (SDQ) [55,56]. The SDQ comprises 25 items that assess 5 domains (5 items each) of well-being as follows: emotional problems, conduct, hyperactivity, peer problems, and prosocial behavior. All but the latter can be summed to generate a total difficulty score [57]. Respondents use a 3-point Likert scale (not true, somewhat true, and certainly true) to indicate the extent to which each item is true for the child on the basis of the child’s behavior over the previous 6 months or over the current school year. The SDQ also includes an impact supplement that assesses the degree to which the child’s difficulties are causing problems in his or her life. Specifically, the teacher rates whether the child has mental health–related difficulties. If the reply is “yes,” then other items examine overall distress, social impairment, burden, and the chronicity of the child’s difficulties.

The questionnaire also included 5 to 6 items, depending on the age of the child, to make a general assessment of the child’s academic skills [58], as well as a question about whether the school provided a general support system for the child.

### Caregiver Questionnaire

#### Overview

Parents, another relative, or another caregiver could fill out the caregiver questionnaire. The contents and wording of the questionnaire were adapted to the type of respondent. As parents were expected to comprise the vast majority of respondents, the following sections use the term parent instead of caregiver. This questionnaire assessed the mental health and well-being of the parent’s child as well as the main known associated factors, such as perinatal factors, lifestyle habits, chronic disease, parenting attitudes, the parent’s mental health, and childhood adversity. The questionnaire was primarily built from standardized and validated questionnaires. It was completed by at least 1 of the parents living with the child every day. For children whose parents were living together, 1 parent was invited to complete it. In other cases, both parents were invited to complete it separately. When several children from the same family were selected, the parent questionnaire was adapted to avoid asking parents the same questions twice regarding their economic situation, sociodemographic characteristics, parent’s mental health, and attitudes on parenting.

#### French Version of the SDQ

Just as for the teacher questionnaire, the French version of the SDQ and the impact supplement were included in the caregiver questionnaire [56].

#### Revised Child Anxiety and Depression Scale (Parent Version)

The parent or caregiver version of the depression subscale of the Revised Child Anxiety and Depression Scale [59-62] was also included for children from first to fifth grades. This subscale comprises 10 items that assess symptoms of the *Diagnostic and Statistical Manual of Mental Disorders, Fourth Edition,* depressive disorder. Each item is rated using a 4-point Likert scale: 0=never, 1=sometimes, 2=often, and 3=always.

#### Autism-Tics, ADHD, and Other Comorbidities Inventory

The caregiver questionnaire also included 5 questions from the Autism-Tics, ADHD, and Other Comorbidities inventory to assess symptoms of autism [63,64] as follows: “Does he/she have difficulties sustaining a conversation?” “Does he/she have difficulty understanding other people’s social cues, e.g., facial expressions, gestures, tone of voice, or body language?” “Does he/she have difficulties behaving as expected by peers?” “Does he/she get absorbed by routines in such a way as to produce problems for him/herself or others?” and “Has he/she ever had a period after the age of 5 when he/she only wanted to eat particular types of food?”

Each question has 3 possible answers, which compare the child with peers during any period of life, as follows: “yes,” “yes to some extent,” and “no.”

#### Childhood Executive Functioning Inventory

The caregiver questionnaire also included 11 items of the Childhood Executive Functioning Inventory to estimate the child’s inhibition functions [65,66]. Answers are based on a 5-point Likert scale, which indicate how well each statement is true for the respondent’s child (definitely not true, not true, partially true, true, or definitely true).

#### French European Developmental Coordination Disorder Questionnaire

The 15 items from the French European Developmental Coordination Disorder Questionnaire were used to evaluate children’s motor coordination in everyday activities compared with that of peers developing typically [67,68]. To keep the questionnaire as brief as possible, only parents with a child in second or third grade were asked to answer these items. Answer options were as follows: “not at all like your child,” “a bit like your child,” “moderately like your child,” “quite a bit like your child,” and “extremely like your child” [67,68].

#### The KINDL Instrument

The KINDL was also included in the caregiver questionnaire to assess the same 6 dimensions of well-being (see the *Child Questionnaire* section) in children in preschool or kindergarten [54]. As was the case for the child questionnaire, the 6-item module on the health-related quality of life for children with chronic illness was also added. Furthermore, the 22 additional items that form the “Kiddy Parents” subscale were included.

#### Self-Harm

Self-harm was evaluated using the following questionnaire item: “Has your child ever tried, in his/her life, to hurt him/herself on purpose?” The following responses were possible: yes, no, do not want to answer, and do not know.

#### Child Health Care Use and Treatments

The questionnaire also explored children’s use of mental health care in the year preceding the study. Specifically, a questionnaire item asked whether the child had been examined for psychological difficulties or specific learning disorders by a general practitioner, a pediatrician, a psychiatrist, a psychologist, a school psychologist, a speech therapist, a pediatric occupational therapist, an orthoptist, a psychometrician, or any other health professional and, for each professional consulted, how many times and where (at the hospital, in private practice, or in a specialized unit) the child had been examined.

Moreover, another item asked whether the child had been prescribed drugs for sleeping, mood, or behavioral disorders during the previous year and, if so, for what reasons from the following list: anxiety, depression, sleeping disorders, inattention problems or hyperactivity, and behavioral problems.

#### Child Medical History

Prenatal (ie, pregnancy complications) and perinatal (ie, neonatal hospitalization and characteristics of the newborn) information was also collected. Furthermore, data on chronic diseases, past hospitalization, mental health history, potentially traumatic events, and significant life events during the previous 12 months were all collected. For this first edition of Enabee (ie, Enabee 2022), questions about COVID-19 exposure (of the child and his or her relatives) were also asked.

#### Family Socioeconomic Characteristics, Home Environment, and Lifestyle of the Child

Parents were asked about family characteristics and living conditions as well as detailed information about their and the other parent’s socioprofessional category, level of education, income (perceived and real), and country of birth (along with the year of arrival in France for those born in another country). Several aspects of the child’s weekly activities and lifestyle were also assessed, including extracurricular time after school, physical activity, screen and social media exposure, sleeping habits, scholastic difficulties, and victimization at school.

#### Parental Health, Social Support, and Parenting Attitudes

The Generalized Anxiety Disorder-7 [69] and the Patient Health questionnaire-9 [70] were used to assess parents’ level of anxiety and depression, respectively. Alcohol, tobacco, and cannabis consumption were explored for both the respondent and the other parent. Respondents were also asked to answer the Global Activity Limitation Indicator single-item indicator for activity limitation for both the respondent and the other parent [71]. Social support was measured using the 3-item Oslo Social Support Scale [72] and loneliness with the 3-item Loneliness Scale [73,74]. In addition, we explored parenting attitudes with the 9-item Alabama Parenting Questionnaire [75] to assess positive parenting, inconsistent discipline, and poor supervision.

### Data Linkage to the French National Administrative Health Care Database

For participants who provided specific consent, data collected in the study will be enhanced, as soon as they become available, with the French national administrative health care database (*Système National des Données de Santé* [SNDS]), which includes data from several databases [76]. Specifically, child data from birth to 18 years of age and data relating to the parent who completed the caregiver questionnaire regarding the 5 years before the mother’s pregnancy until the date of the Enabee study will be matched with data from Enabee.

One of the databases covered by the SNDS is the French health insurance database (*Données de consommation inter-régimes*). The *Données de consommation inter-régimes* contains an exhaustive record of all outpatient visits, prescriptions, and reimbursements for out-of-pocket health care spending for over 99% of the population living in France. Reimbursable drugs are coded using the Anatomical Therapeutic Chemical classification. Medical and surgical procedures, prescribed medical devices, and biological examinations are all recorded according to the Common Classification of Medical Procedures (*Classification Commune des Actes Medicaux*).

Another database covered by the SNDS is the *Programme de médicalisation des systèmes d’information*, which is a database providing admission and discharge information for all public and private hospital stays throughout the French territory. All diagnoses in the *Programme de médicalisation des systèmes d’information* are coded according to the *International Classification of Diseases, 10th Edition*, while the main medical and surgical procedures performed are coded according to the *Classification Commune des Actes Medicaux*. Chaining Enabee data and SNDS data will allow us to link the declarative data from the questionnaires to present and future (reimbursed) mental health care use.

### Statistical Analyses

To produce national estimates of children’s mental health, statistical analyses will take into account the sampling design of the study, including nonresponse weight adjustment, strata, and the finite population correction factor. Estimates of children’s mental health will be provided by an informant (child, teacher, and caregiver). Methods combining answers from multiple informants, which will produce more reliable predictions of psychiatric disorders than from single-informant scores [77,78], will be used.

### Ethical Considerations

The design of the study was discussed by a steering committee and by a scientific advisory board. A consultation group, bringing together the main stakeholders in the fields of health, education, and family, discussed the best possible ways to successfully conduct the study.

The first edition of Enabee (ie, Enabee 2022) was approved by the French authority for data protection (*Commission nationale informatique et libertés*, deliberation DR-2022-009 of January 7, 2022), as well as by a French ethics committee (*Comité éthique et scientifique pour les recherches, les études et les évaluations dans le domaine de la santé*, decision n° 5268850, October 14, 2021). The survey also received approval from the French National Council for Statistical Information (avis n° 85/H030) and the Committee of Public Statistics (N° 2022_11193_DG75-L002). Personal data processing was performed in compliance with the European Union’s General Data Protection Regulation. For parents and teachers, a free phone support hotline (including psychologists) was open during the full period of the study for any questions or comments related to the study or to mental health.

Following a specific request by *Comité éthique et scientifique pour les recherches, les études et les évaluations dans le domaine de la santé*, an algorithm to identify children who might need to be referred to their school health service for an evaluation of their mental health was created based on children’s responses to the DI (see the *Child Questionnaire* section). Specifically, children whose answers indicated that they presented probable depression and at least 1 probable externalizing disorder (ADHD or oppositional defiant disorder) *and* who answered “yes” for both the 2 questions on thoughts about death and suicidal thoughts were identified as possibly needing referral. When the algorithm identified such a child, a trusted third party transferred the name of the child to the school health service while respecting medical confidentiality.

## Results

### Pilot Study

A pilot study was conducted in January 2022 to test the Enabee protocol, in particular, its feasibility, acceptability, data collection procedures, and participation rates, and to identify potential areas for improvement. Despite the COVID-19 pandemic, the pilot study was successfully implemented in 20 schools, representing 93 classes and 1977 pupils. Feedback showed that school principals, teachers, and parents were all interested in the study and its topic. Children participating in the pilot study were also very happy to have had an “out-of-the-ordinary activity that they enjoyed” (study facilitator). The school participation rate for this study was 95% (19/20). Among the schools that agreed to take part, 3% (3/86) of the classes subsequently did not participate because of the teachers’ absence on the day of the study. Among teachers, 76% (65/86) completed the questionnaire for all their pupils. A small proportion of parents (36/1977, 1.82%) were opposed to their child’s participation and 3.49% (64/1835) of the parents did not receive study information at home. Among parents who agreed to participate, 35.69% (632/1771) fully completed the caregiver questionnaire. The child questionnaire was completed by 82.54% (936/1134) of the children solicited to participate. Of the remaining 18.34% (208/1134), 14.37% (163/1134) were absent the day of the survey (mainly because of COVID-19), 3.09% (35/1134) refused to answer the questionnaire, and 0.88% (10/1134) did not finish it.

To foster parent participation, various adjustments were made to the protocol after the pilot study. First, communication was improved to give enough time for school principals and teachers to better inform the pupils’ parents about the study. Second, changes were made to reduce the average time needed to complete the caregiver questionnaire from 51 minutes to 45 minutes. Other adjustments were made to improve the study information letters, facilitator training, clarity of the child questionnaire, and clarity of the study website.

### First Edition: Enabee 2022

Data were collected for Enabee 2022 from May 2, 2022, to July 31, 2022. Data analysis is still ongoing. Among the 706 sampled schools, 399 (56.5%) agreed to participate, representing 1464 classes. No difference in participation was observed between private schools, public schools located outside socially deprived areas, and public schools located in socially deprived areas. The main reason cited for nonparticipation was the lack of time to implement the study.

Among the 1464 classes drawn for participation, 1357 (92.69%) teachers agreed for their class to participate, representing 29,389 children. After excluding children who moved house (169/29,389, 0.58%), children whose parents objected to participation (2127/29,389, 7.24%), children whose parents did not receive study information at home (1818/29,389, 6.19%), and children whose teacher considered that they were not able to properly complete the child questionnaire due to a disability or too poor a French linguistic level (questionnaires for these children were completed but will be analyzed separately; 567/29,389, 1.93%), 24,708 pupils from preschool to fifth grade were considered for participation (24,708/29,389, 84.07%; ie, 8271 pupils in preschool or kindergarten and 16,437 pupils from first to fifth grades). A teacher questionnaire was completed for 5721 (69.17%) children of the preschool or kindergarten pupils and a caregiver questionnaire for 3785 (45.76%) children. For the pupils from first to fifth grades, a teacher questionnaire was completed for 92.86% (15,263/16,437) children, a caregiver questionnaire for 56.14% (9227/16,437) children, and a child questionnaire for 92.51% (15,206/16,437) children. On the basis of the answers to the DI, 391 children were addressed to the school health service for an evaluation of their mental health (ie, 2.6% of all those who answered the child questionnaire). The first published results in peer-reviewed journals are expected in 2024.

## Discussion

### Principal Findings

Before the development of the Enabee surveillance system, data on children’s mental health were scarce in France. The collaborative conception and the successful implementation of the Enabee 2022 study demonstrate the feasibility and acceptability of this school-based, cross-sectional study evaluating children’s mental health in metropolitan France.

The Enabee 2022 study, as part of the Enabee surveillance system, will provide, for the first time in France, nationwide surveillance data on the mental health and well-being of children aged 3 to 11 years. Many factors, including socioeconomic characteristics, the child’s home environment, lifestyle, and parents’ mental health, were measured in the caregiver questionnaire to study associations with mental health disorders and level of well-being. These data are relevant for national authorities to develop data-driven public health policies.

In addition to the orientation of these policies, the Enabee surveillance system could be used to follow the short- and long-term effects of a future health crisis similar to the COVID-19 pandemic or other events (terrorist attacks, extreme weather events, major economic crises, etc) that are known to impact children’s mental health [79], with the view of providing mitigation recommendations. As Enabee uses internationally recognized psychometric scales, it will provide reference data for international and temporal comparisons, while nationwide comparison data will be available for other research studies on specific subpopulations in France.

### Strengths and Limitations

The Enabee protocol has limitations inherent to studies on mental health and to surveillance systems that use psychometric scales to assess children’s mental health and well-being. First, mental health symptoms in children fluctuate over time, and both symptoms and their reporting can be influenced by recent experiences. In this context, the scales used in Enabee may inflate mental disorder prevalence rates, as the distinction between psychological disorders and psychological distress can be difficult to establish [80]. However, the “impact supplement” in the SDQ may reduce this bias.

Second, nonresponse from some schools, children, parents, and teachers may generate selection bias. However, considering the sophisticated sampling design (survey weights, stratification, and sampling stages) used, it seems reasonable to suppose that the potential impact of selection bias will be limited.

Third, because of the sensitivity of the study topic, social desirability bias may influence the answers. However, Schlenger and Silver [81] pointed out that web-based data collection appears to reduce this kind of bias.

Fourth, as Enabee is a national study, there will be insufficient numbers of children from very specific populations, such as children who are homeless and children in child welfare, to support analyses on those specific populations. However, the sampling design was conceived to overrepresent children from deprived circumstances; this will allow us to analyze social health inequities related to children’s mental health.

Fifth, the surveillance system’s mental health coverage is incomplete. In particular, there is no comprehensive survey of neurodevelopmental disorders. While ADHD and developmental coordination disorder are well covered, only 5 questions focus on autism spectrum disorder. Items on intellectual disabilities, communication, and learning disorders are absent. This is, in part, due to the impossibility of administering comprehensive tests on these disorders to children participating in the first edition of Enabee, as they are too demanding.

Finally, Enabee 2022 was developed under time constraints. The relatively short development time frame (1 year) meant that we were not able to add a voice over or to translate the caregiver questionnaire into languages other than French, making the participation of non–French speaking parents difficult. Furthermore, there was insufficient time to create adjustments to meet the specificities of France’s 5 overseas territories (Martinique, Guadeloupe, French Guiana, Reunion Island, and Mayotte). This will have to be done before Enabee can be implemented there. Accordingly, data for these territories will be collected at a later date.

The protocol also has strengths. First, unlike other surveillance systems and most of the epidemiological studies on children, which have data from only 1 informant, Enabee is based on data from 2 to 3 informants, depending on the age of the child. This ensures that different perspectives can be compared and integrated. Most importantly, Enabee takes into account the perspective of children from first to fifth grades. This is particularly important in the context of assessing emotional disorders, an area where children are recognized as being good informants; indeed, they report such problems more frequently than parents [82]. Children are interviewed at school during school time, ensuring homogenous conditions for answering the questionnaires and reducing bias related to the way the questionnaire is administered.

Second, participation rates in household surveys have been decreasing worldwide since the 1990s [20,83]. We believe that our school-based design may lead to greater participation for children and teachers and, therefore, greater participation by parents. Indeed, in the first edition of Enabee in 2022, we observed greater participation by parents of children in grades 1 to 5 who agreed to answer the child questionnaire than by parents of children in preschool or kindergarten (ie, no child questionnaire completed; 9227/16,437, 56.14% vs 3785/8271, 45.76%).

Third, the study was designed and implemented after broad consultation between stakeholders and experts to obtain as high a participation rate as possible over the upcoming years when the study is repeated.

Finally, the pilot study provided us with useful information on how to improve the protocol and**,** especially**,** to increase parent participation (from 632/1771, 35.69% in the pilot study to 13,012/24,708, 52.66% in Enabee 2022).

### Conclusions

The first edition of the French Enabee study, as part of the homonymous surveillance system, was successfully conducted in 2022. It had one of the largest national random samples of any study on children’s mental health worldwide to date and was the first national study in metropolitan France to assess mental health in children aged between 3 and 11 years. This sample size will ensure good precision in our estimates and allow us to conduct analyses with sufficient statistical power. As Enabee will be repeated over time, in future editions, it will be possible to explore additional issues to have a more comprehensive picture of children’s mental health in France. The COVID-19 pandemic highlighted the importance of mental health surveillance in children and young persons. In the context of the launch of the 2022-2037 French interministerial strategy for the development of social emotional learning among children and young people [84], stakeholders are eagerly awaiting the results of Enabee 2022, as they will constitute one of the essential elements in the development of public health policies to improve the mental health of children in France and to facilitate access to mental health care when needed.

## Appendix

Multimedia Appendix 1 Example of a Dominic Interactive question: “Do you worry a lot about your parents having a car accident, like Dominic?” Answer: yes or no.

Multimedia Appendix 2 Example of a KINDL question: Now tell us about how you feel: “During the past week, I laughed a lot and I had lots of fun.” Answers: never, rarely, sometimes, often, and always.

## Abbreviations

ADHD: attention-deficit/hyperactivity disorder
DI: Dominic Interactive
Enabee: Etude nationale sur le bien-être des enfants
Fdep: French Deprivation Index
SDQ: Strengths and Difficulties Questionnaire
SNDS: Système National des Données de Santé

## Data Availability

The data sets analyzed during this study are available from the corresponding author upon reasonable request.
