# Supplementary material for: Implementation of a Novel Epidemiological Surveillance System for Children’s Mental Health and Well-Being in France: Protocol for the National “Enabee” Cross-Sectional Study
Source: JMIR Public Health Surveill. 2024 Aug 13;10:e57584. doi: 10.2196/57584 (PMC11350310; doi:10.2196/57584)

## Multimedia Appendix 1

Example of a DI question: (translated from French) “Do you worry a lot about your parents having a car accident, like Dominic?” (note: Dominic is a unisex name in France) Answer: Yes / No, Enabee 2022


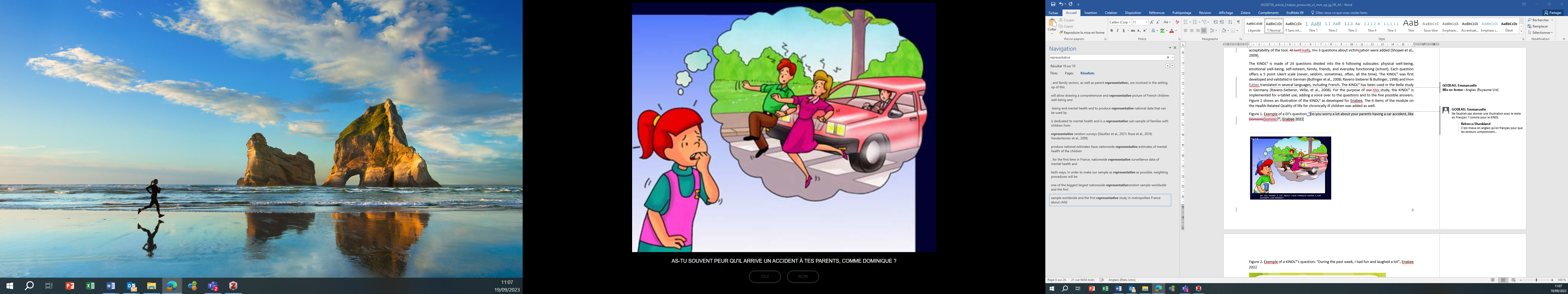


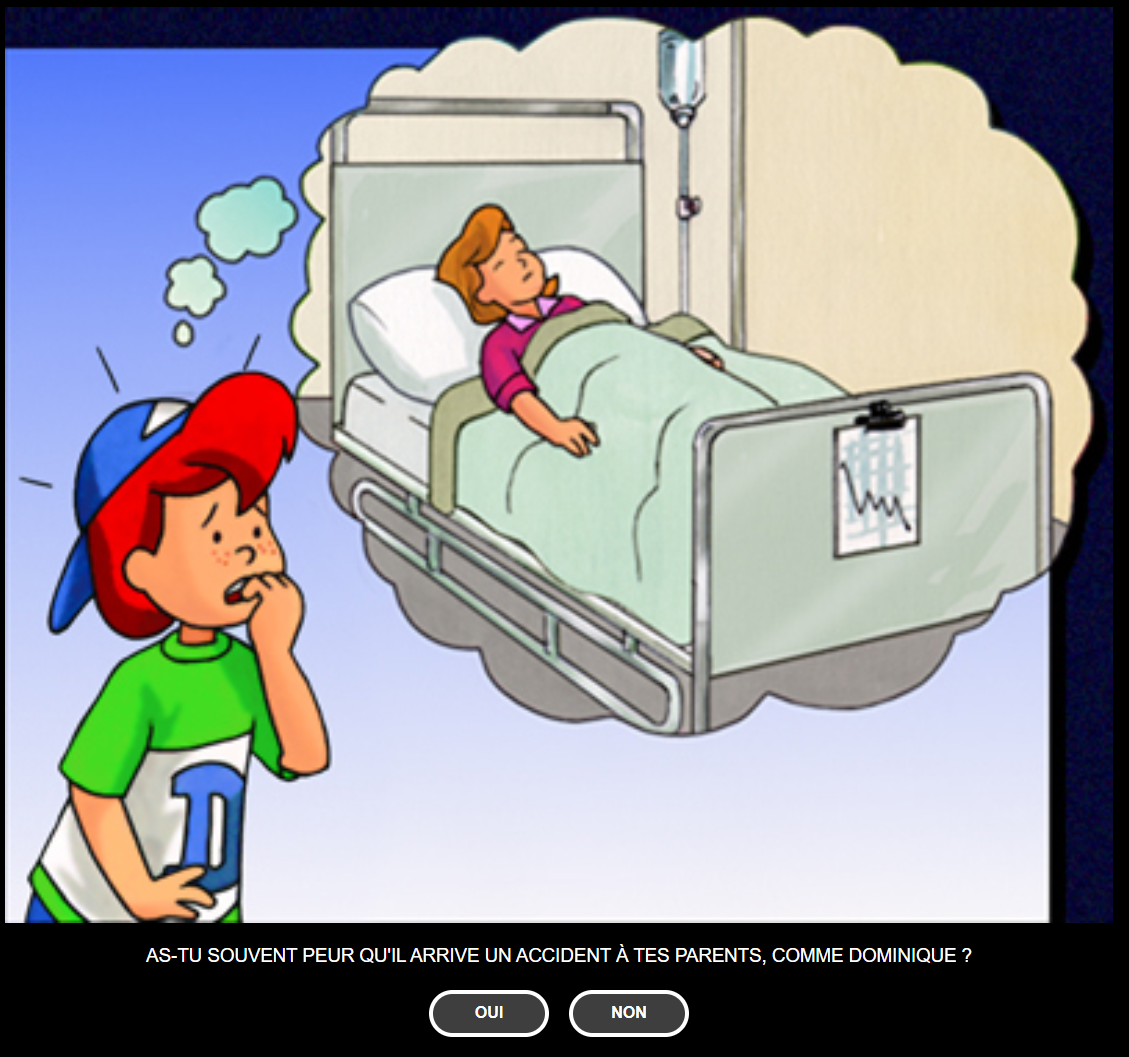

Supplement: Multimedia Appendix 1 [file publichealth_v10i1e57584_app1.docx]
