# Supplementary material for: Implementation of a Novel Epidemiological Surveillance System for Children’s Mental Health and Well-Being in France: Protocol for the National “Enabee” Cross-Sectional Study
Source: JMIR Public Health Surveill. 2024 Aug 13;10:e57584. doi: 10.2196/57584 (PMC11350310; doi:10.2196/57584)

## Multimedia Appendix 2

Example of a KINDL^R^ question: Now tell us about how you feel: “During the past week, I laughed a lot and I had lots of fun”. Answers: Never, Rarely, Sometimes, Often, Always, Enabee 2022


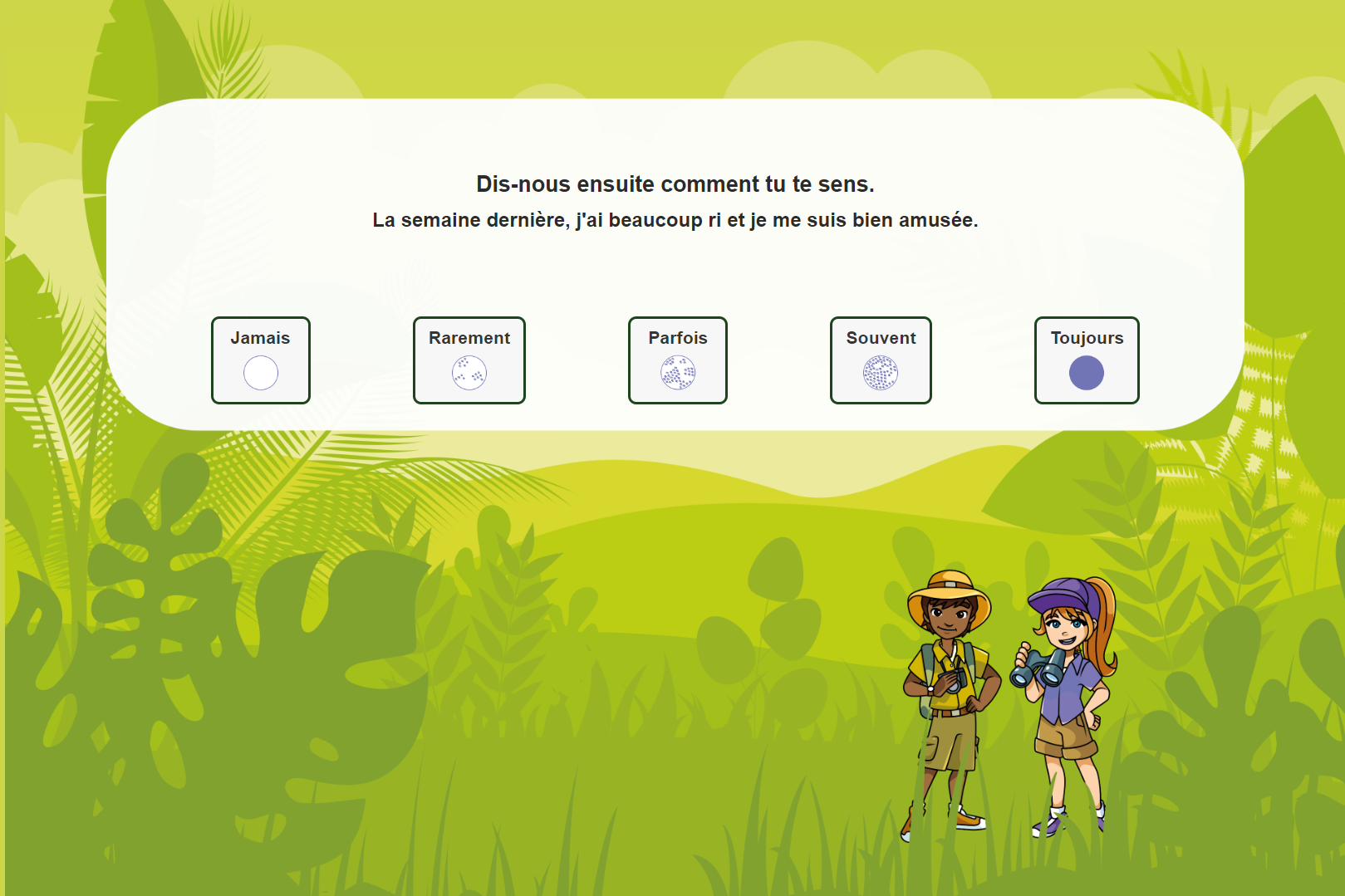

Supplement: Multimedia Appendix 2 [file publichealth_v10i1e57584_app2.docx]
